# Million Hearts: Description of the National Surveillance and Modeling Methodology Used to Monitor the Number of Cardiovascular Events Prevented During 2012–2016

**DOI:** 10.1161/JAHA.117.006021

**Published:** 2017-05-02

**Authors:** Matthew D. Ritchey, Fleetwood Loustalot, Hilary K. Wall, Claudia A. Steiner, Cathleen Gillespie, Mary G. George, Janet S. Wright

**Affiliations:** ^1^ Division for Heart Disease and Stroke Prevention Centers for Disease Control and Prevention Atlanta GA; ^2^ Agency for Healthcare Research and Quality Rockville MD

**Keywords:** cardiovascular disease prevention, heart disease, hospitalization, Million Hearts, mortality, myocardial infarction, stroke, Epidemiology, Quality and Outcomes, Cerebrovascular Disease/Stroke, Mortality/Survival, Myocardial Infarction

## Abstract

**Background:**

This study describes the national surveillance and modeling methodology developed to monitor achievement of the Million Hearts initiative's aim of preventing 1 million acute myocardial infarctions, strokes, and other related cardiovascular events during 2012–2016.

**Methods and Results:**

We calculate sex‐ and age‐specific cardiovascular event rates (combination of emergency department, hospitalization, and death events) among US adults aged ≥18 from 2006 to 2011 and, based on log‐linear models fitted to the rates, calculate their annual percent change. We describe 2 baseline strategies to be used to compare observed versus expected event totals during 2012–2016: (1) *stable baselines* assume no rate changes, with modeled 2011 rates held constant through 2016; and (2) *trend baselines* assume 2006–2011 rate trends will continue, with the annual percent changes applied to the modeled 2011 rates to calculate expected 2012–2016 rates. Events prevented estimates during 2012–2013 were calculated using available data: 115 210 (95% CI, 60 858, 169 562) events were prevented using stable baselines and an excess of 43 934 (95% CI, −14 264, 102 132) events occurred using trend baselines. Women aged ≥75 had the most events prevented (stable, 76 242 [42 067, 110 417]; trend, 39 049 [1901, 76 197]). Men aged 45 to 64 had the greatest number of excess events (stable, 22 912 [95% CI, 855, 44 969]; trend, 38 810 [95% CI, 15 567, 62 053]).

**Conclusions:**

Around 115 000 events were prevented during the initiative's first 2 years compared with what would have occurred had 2011 rates remained stable. Recent flattening or reversals in some event rate trends were observed supporting intensifying national action to prevent cardiovascular events.

## Introduction

In the US, cardiovascular disease (CVD) accounts for more than $300 billion in annual costs.^1^ A major cause of disability^2^ and leading cause of death, CVD accounts for nearly 30% of all deaths—more than 800 000 deaths each year.^3^ Many CVD‐related events, including emergency department (ED) visits, hospitalizations, and deaths, are avoidable, given that they can be attributed to a lack of healthy lifestyle behaviors, preventive health care, or timely and effective medical care.^1^, ^4^, ^5^, ^6^, ^7^ In recent decades, the United States has experienced continuous declines in cardiovascular event rates.^1^, ^8^ However, because of an aging population^9^ and the high prevalence of CVD risk factors,^1^ increased CVD prevalence and a slowing or reversal in previously declining overall cardiovascular event rate trends have been reported up through 2011 and forecast to continue if additional efforts are not taken.^10^, ^11^, ^12^, ^13^ Some have suggested that the United States needs to establish and take action on achieving aggressive targets for CVD risk factor prevention and management and continue to improve adherence to evidence‐based treatments to make substantial gains in decreasing the CVD burden.^14^, ^15^, ^16^

In response, the US Department of Health and Human Services launched the Million Hearts initiative in 2012 to focus, coordinate, and enhance CVD prevention activities across the public and private sectors to prevent 1 million acute myocardial infarctions (AMIs), strokes, and other related cardiovascular events by 2017.^17^ Million Hearts is working to meet this goal by focusing its efforts on the ABCS of clinical care—**a**spirin, when appropriate, for secondary CVD prevention; **b**lood pressure control; **c**holesterol management; and **s**moking cessation—and in the community setting by reducing overall tobacco use, reducing sodium consumption, and eliminating artificial trans fat from the diet. These intermediate measures have been tracked since Million Hearts began and will continue to be monitored.^18^, ^19^ Population‐level improvements in these risk factors have been demonstrated to lead to decreases in cardiovascular events.^14^, ^17^, ^20^, ^21^, ^22^

A national surveillance system to collectively track nonfatal and fatal cardiovascular events in the United States is not available, limiting the ability to monitor the effects of the initiative on event totals.^23^ The purpose of our study is to describe how Million Hearts is using a novel method to combine ED, hospitalization, and mortality data sets to overcome this limitation in national cardiovascular event surveillance and assess whether the initiative met the aim of preventing 1 million events. Using this methodology, we describe the burden of Million Hearts events before the initiative (2006–2011) and 2 strategies used to predict the expected number of events that would occur during the initiative (2012–2016) without additional intervention and estimate the number of events potentially prevented. We provide events prevented estimates for 2012 and 2013 using currently available data.

## Methods

### Description of Data Sources

We used encounter‐level data from the Agency for Healthcare Research and Quality's Healthcare Cost and Utilization Project's Nationwide Emergency Department Sample^24^ (NEDS) to capture ED events; annually, it contains 25 to 30 million unweighted ED visit records from over 950 sampled hospitals. We used the provided NEDS weights to calculate national ED estimates and SDs based on the survey's complex sampling design. We used encounter‐level data from the Healthcare Cost and Utilization Project's Nationwide Inpatient Sample^25^ to capture hospitalization events during 2006 to 2011; annually, it contains ≈8 million unweighted hospital stays from ≈1000 sampled hospitals. Beginning in 2012, the Nationwide Inpatient Sample was redesigned^26^ as the National Inpatient Sample (collectively referred to throughout as the NIS), selecting a sample of discharges from all hospitals participating in the Healthcare Cost and Utilization Project's, which represents more than 95% of the US population and provides an almost population‐level account of inpatient utilization. We used the Agency for Healthcare Research and Quality's NIS trend file^27^ to establish the hospitalization trend data for 2006–2011 to adjust for the sampling methodology change between 2011 and 2012.

We used mortality data from the Centers for Disease Control and Prevention's National Vital Statistics System (NVSS) Mortality Multiple Cause of Death Files to calculate national mortality estimates and SEs that account for non‐sampling‐related errors in the death reporting process.^28^ The NVSS collects information reported on all death certificates filed in every US state and the District of Columbia.

### Identification of Million Hearts Events

**Figure 1:**
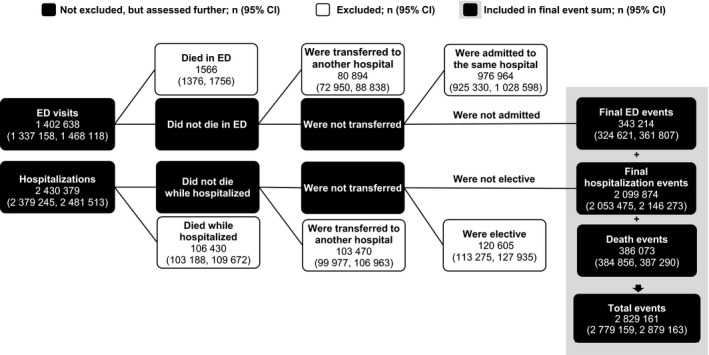
Million Hearts mutually exclusive event calculation methodology applied to the 2013 Nationwide Emergency Department Sample, National Inpatient Sample, and National Vital Statistics System data to determine the total number of mutually exclusive events. After applying the exclusion criteria, 343 214 ED visits, 2 099 874 hospitalizations, and 386 073 deaths were summed for a total of 2 829 161 mutually exclusive events that occurred in 2013. ED indicates emergency department. Additional details are included in Figure S1.

The events case definition was developed based on subject‐matter expert input, literature findings, and past coding practices. Multiple decision points were considered in development of the definition (see Data S1), with the most important criterion for an event type's inclusion being that it could be expected to be prevented by current or planned Million Hearts efforts. This led to a definition that included a broader range of event types (eg, included heart failure) than only AMIs and strokes. Although effective from a communications standpoint, the name “Million Hearts” and the aim “to prevent 1 million heart attacks and strokes”^17^ are limited in conveying the full complement of the events targeted and assessed. We defined a Million Hearts event as a CVD‐related ED encounter or hospitalization with a primary diagnosis International Classification of Diseases, Ninth Revision, Clinical Modification code^29^ (specified in Table S1) or a death with an International Classification of Diseases, Tenth Revision underlying cause of death code^30^ (specified in Table S1) among adults aged ≥18 years during 2006–2011. We then applied exclusion criteria to each data set to estimate the number of mutually exclusive acute events that were captured in each data set each year (Figure 1; additional details provided in Figure S1). This involved excluding ED visit events where the patients died in the ED, were transferred to another hospital, or were admitted to the same hospital and excluding hospitalization events that were reported as elective or where the patient died in the hospital or was transferred to another hospital. Million Hearts event totals were then summed across the data sets and SDs calculated (see Data S1). Events were assigned to 1 of 4 categories to describe their distribution—AMIs; strokes; symptomatic precursor conditions (eg, stable angina pectoris); or other CVD conditions (eg, heart failure). The combined totals represent the events tracked by the initiative.

### Statistical Analysis

#### Calculation of event rates and assessment of trends

We calculated annual Million Hearts events rates, stratified by sex (men or women; the NEDS and NIS collects this information as sex and the NVSS as gender) and age group (18–44, 45–64, 65–74, or ≥75 years). The ranges for the age groups align with those reported for Million Hearts’ risk factor measures.^19^ Race/ethnicity‐specific rates were not calculated because race/ethnicity is not consistently included within the NEDS and NIS. We calculated rates and their SDs, using intercensal population estimates as the demoninators,^9^, ^31^ and age‐standardized the overall and sex‐specific rates to the 2010 US Decennial Census population,^31^ using the age categories listed above.

We characterized temporal trends in event rates during 2006–2011 by fitting log‐linear regression models to the sex‐ and age‐specific rates, weighted by the inverse of each rate's variance. We used the log‐transformed regression coefficients (slopes) of the event rate models to calculate the annual percent change (APC) for each rate and the associated SEs and 95% CIs.^32^ We tested the APCs within each sex and age group against the null hypothesis (slope=0); we interpreted models with APCs that were statistically different from 0 as having a change in event rate during the period (*P*<0.05). We compared APCs across age groups for each sex (referent, adults aged 18–44 years) and in each age group by sex (referent, men) to test for statistically significant differences (*P*<0.05). All statistical tests were 2‐sided and conducted using SAS software (9.3; SAS Institute Inc, Raleigh, NC). This study was exempt from institutional review board review because the data analyzed were from de‐identified, public‐use data sets made available for public health surveillance.

#### Development and use of two baseline strategies

Two parallel baseline strategies were developed to assess the association between the implementation of Million Hearts and decreases in event totals during 2012–2016. The first strategy (stable baselines) assumes there will be no change in event rates during 2012–2016 unless additional action is taken. To establish the stable baselines, we held the modeled sex‐ and age‐specific 2011 rates constant during 2012–2016. The second strategy (trend baselines) assumes the event rate trends experienced during 2006–2011 will continue during 2012–2016. To establish the trend baselines, we applied the sex‐ and age‐specific APCs to the modeled 2011 rates to calculate expected rates for 2012–2016 (see Data S1 and Figure S2 for an example). We applied the sex‐ and age‐specific modeled 2011 rates and, for the trends baseline, the sex‐ and age‐specific APCs and their associated SEs, to generate 1000 simulated expected rate estimates for each baseline strategy. We then applied the means and SDs of the simulated rates from both baseline strategies against the US Census population estimates^9^, ^31^ to determine the mean number of events, including the associated 95% CIs, expected to occur each year according to both strategies.

NIS, NEDS, and NVSS data were available for 2012 and 2013, so observed event rates and totals were calculated for those years. To estimate the number of events prevented during the first 2 years of the initiative, we subtracted the mean number of expected events obtained from the simulations from the number of actual events observed each year. These differences were then assessed annually and in combination for both years to see whether they were statistically significantly different than 0 (*z*‐score ≥|1.96|). Events prevented estimates, including the use of revised population estimates, will be updated by Million Hearts as data become available for 2014 to 2016.

## Results

### Event Rates Preceding the Initiative

**Figure 2:**
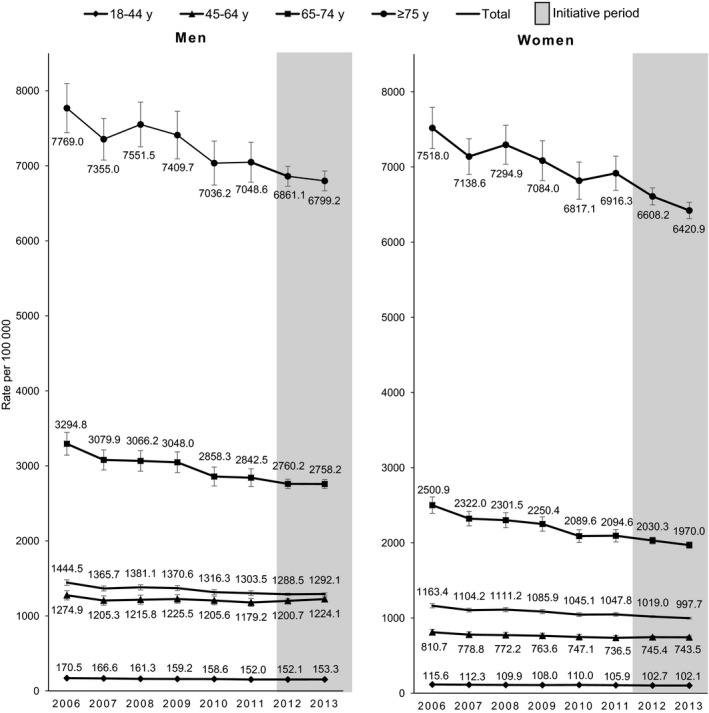
Million Hearts event rates before and during the Million Hearts initiative, by sex and age group, 2006–2013. Total rates were standardized by age to the 2010 US population. Bars denote 95% confidence intervals for the rates.

Prior to the beginning of the initiative, the age‐standardized event rate (per 100 000) among all adults decreased 9.8% from 1296.8 (95% CI, 1265.9, 1327.8) in 2006 to 1170.3 (95% CI, 1144.7, 1195.9) in 2011 (Table 1), with similar relative percent decreases in the age‐standardized event rates observed among men (9.8%) and women (9.9%; Figure 2). Every age group among both sexes experienced statistically significant declines in event rates during 2006 to 2011 (*P*<0.05), except for men aged 45 to 64 years (Table 2). The annual event rate decreased at a statistically significant greater pace among women aged 65 to 74 years compared with women aged 18 to 44 years (*P*=0.002); it decreased at a statistically significant slower pace among men aged 45 to 64 years compared with men aged 18 to 44 years (*P*=0.033).

**Table 1:** Age‐Standardized Rates^a^ by Event Category and Event Type, 2006–2013

Event Category or Type	Age‐Standardized Rate Per 100 000 (95% CI)							
	2006	2007	2008	2009	2010	2011	2012	2013
Total	**1296.8 (1265.9, 1327.8)**	**1228.6 (1200.4, 1256.8)**	**1239.5 (1210.1, 1268.9)**	**1220.9 (1191.5, 1250.2)**	**1174.5 (1146.7, 1202.3)**	**1170.3 (1144.7, 1195.9)**	**1147.6 (1134.6, 1160.6)**	**1137.6 (1125.0, 1150.2)**
AMI	**284.8 (276.7, 292.8)**	**260.2 (253.3, 267.1)**	**269.0 (262.0, 275.9)**	**261.9 (254.8, 269.1)**	**244.6 (237.9, 251.4)**	**244.4 (238.3, 250.5)**	**249.3 (245.8, 252.8)**	**243.9 (240.6, 247.1)**
Stroke	**242.1 (238.2, 246.1)**	**235.4 (231.7, 239.1)**	**243.2 (239.2, 247.3)**	**234.6 (230.6, 238.6)**	**241.3 (237.2, 245.4)**	**243.7 (239.5, 247.9)**	**241.4 (239.1, 243.7)**	**241.4 (239.1, 243.7)**
Acute ischemic cerebral infarction	157.0 (153.3, 160.7)	153.3 (149.8, 156.8)	161.8 (157.9, 165.7)	156.8 (152.9, 160.6)	161.5 (157.6, 165.4)	167.7 (163.7, 171.6)	166.9 (164.7, 169.0)	167.5 (165.4, 169.6)
Acute hemorrhagic stroke	36.5 (35.3, 37.7)	34.5 (33.3, 35.6)	35.1 (33.9, 36.2)	34.2 (33.0, 35.3)	36.7 (35.4, 37.9)	33.7 (32.3, 35.0)	33.2 (32.5, 33.9)	33.2 (32.5, 33.9)
Acute, but ill‐defined, cerebrovascular disease	1.0 (0.9, 1.2)	0.8 (0.7, 0.9)	0.7 (0.6, 0.8)	0.5 (0.4, 0.7)	0.4 (0.3, 0.4)	0.3 (0.3, 0.4)	0.2 (0.2, 0.3)	0.2 (0.1, 0.2)
Other cerebrovascular disease deaths	47.6 (47.3, 47.9)	46.9 (46.6, 47.2)	45.7 (45.4, 45.9)	43.1 (42.9, 43.4)	42.8 (42.5, 43.0)	42.0 (41.8, 42.3)	41.1 (40.9, 41.4)	40.5 (40.3, 40.8)
Symptomatic precursor conditions	**184.0 (180.9, 187.0)**	**171.4 (168.7, 174.1)**	**169.5 (166.6, 172.4)**	**161.0 (158.1, 164.0)**	**151.2 (148.6, 153.8)**	**150.4 (147.9, 152.9)**	**146.5 (144.6, 148.4)**	**139.7 (137.8, 141.6)**
Other acute and subacute ischemic heart disease	33.3 (32.1, 34.4)	29.1 (28.1, 30.1)	25.4 (24.6, 26.3)	24.1 (23.2, 24.9)	21.1 (20.3, 21.9)	20.3 (19.5, 21.2)	19.9 (19.3, 20.4)	19.7 (19.1, 20.3)
Stable angina pectoris	24.6 (23.8, 25.3)	22.7 (22.0, 23.4)	21.1 (20.3, 21.8)	19.0 (18.4, 19.6)	17.8 (17.2, 18.4)	16.7 (16.1, 17.3)	16.2 (15.6, 16.7)	15.4 (14.9, 16.0)
Transient ischemic attack	126.1 (123.3, 128.8)	119.6 (117.3, 122.0)	123.0 (120.3, 125.7)	117.9 (115.2, 120.7)	112.2 (109.8, 114.6)	113.4 (111.1, 115.7)	110.5 (108.8, 112.2)	104.5 (102.8, 106.3)
Other CVD conditions	**586.0 (575.5, 596.4)**	**561.6 (551.9, 571.4)**	**557.8 (548.1, 567.5)**	**563.3 (553.4, 573.1)**	**537.4 (527.9, 546.8)**	**531.8 (523.3, 540.3)**	**510.4 (505.9, 515.0)**	**512.6 (508.1, 517.1)**
Heart failure	512.8 (502.5, 523.1)	480.8 (471.2, 490.3)	477.0 (467.5, 486.5)	479.4 (469.7, 489.0)	453.4 (444.1, 462.6)	449.0 (440.7, 457.3)	428.1 (423.6, 432.5)	434.4 (430.0, 438.8)
Abdominal aortic aneurysms	1.9 (1.7, 2.0)	1.7 (1.6, 1.9)	2.0 (1.8, 2.1)	1.7 (1.5, 1.8)	1.7 (1.5, 1.8)	1.5 (1.4, 1.7)	1.5 (1.4, 1.6)	1.5 (1.4, 1.6)
Atheroembolism	0.5 (0.4, 0.5)	0.4 (0.3, 0.4)	0.4 (0.3, 0.4)	0.4 (0.3, 0.4)	0.4 (0.3, 0.4)	0.3 (0.3, 0.4)	0.4 (0.3, 0.4)	0.3 (0.3, 0.3)
Atherosclerosis and peripheral artery disease	7.0 (6.9, 7.1)	6.6 (6.5, 6.7)	6.3 (6.2, 6.4)	6.0 (5.9, 6.1)	5.9 (5.8, 6.0)	5.6 (5.5, 5.7)	5.6 (5.5, 5.7)	5.5 (5.4, 5.6)
Hypertension without heart failure	63.4 (61.5, 65.3)	71.6 (69.7, 73.6)	71.7 (69.7, 73.8)	75.5 (73.3, 77.6)	75.7 (73.8, 77.5)	75.0 (73.3, 76.8)	74.7 (73.7, 75.6)	70.7 (69.8, 71.6)
Cardiac arrest	0.5 (0.4, 0.6)	0.5 (0.5, 0.6)	0.5 (0.4, 0.5)	0.4 (0.4, 0.5)	0.4 (0.4, 0.5)	0.3 (0.2, 0.3)	0.3 (0.2, 0.3)	0.3 (0.3, 0.4)

a Standardized by age to the 2010 US Census Population distribution among adults aged ≥18 years. AMI indicates acute myocardial infarction; CVD, cardiovascular disease.

**Table 2:** Changes in Sex‐ and Age‐Specific Event Rates During 2006–2011 and Expected Rates Using Both Baseline Strategies

Sex	Age Group (y)	APC During 2006–2011 (95% CI)	Stable Baseline Rate for 2012–2016^a^	Trend Baseline Rates, Per 100 000, for 2012–2016 (95% CI)^b^				
			Rate Per 100 000 (95% CI)	2012	2013	2014	2015	2016
Men	18 to 44	−2.1 (−2.7, −1.5)	152.9 (150.9, 154.9)	149.7 (147.5, 151.9)	146.5 (144.1, 148.9)	143.4 (140.7, 146.1)	140.3 (137.2, 143.4)	137.3 (133.6, 141.0)
	45 to 64	−1.1^c^ (−2.2, 0.0)	1184.1 (1156.5, 1211.7)	1171.0 (1143.4, 1198.6)	1158.0 (1126.4, 1189.6)	1145.2 (1108.0, 1182.4)	1132.5 (1088.4, 1176.6)	1119.9 (1068.4, 1171.4)
	65 to 74	−2.7 (−3.9, −1.4)	2827.4 (2753.1, 2901.7)	2752.1 (2676.2, 2828.0)	2677.9 (2592.8, 2763.0)	2605.8 (2507.0, 2704.6)	2535.7 (2421.2, 2650.2)	2467.5 (2336.4, 2598.6)
	≥75	−1.7 (−3.1, −0.4)	7031.0 (6823.6, 7238.4)	6909.2 (6694.4, 7124)	6788.5 (6547.6, 7029.4)	6670.0 (6391.1, 6948.9)	6553.8 (6230.4, 6877.2)	6439.8 (6068.8, 6810.8)
Women	18 to 44	−1.4 (−2.4, −0.5)	106.3 (104.1, 108.5)	104.8 (102.6, 107.0)	103.2 (100.7, 105.7)	101.7 (98.8, 104.6)	100.3 (96.8, 103.8)	98.8 (94.7, 102.9)
	45 to 64	−1.7 (−2.2, −1.2)	735.1 (727.5, 742.7)	722.4 (714.4, 730.4)	709.9 (700.9, 718.9)	697.6 (687.0, 708.2)	685.5 (673.0, 698.0)	673.6 (659.1, 688.1)
	65 to 74	−3.4^d^ (−4.7, −2.0)	2069.8 (2010.6, 2129.0)	1999.9 (1939.1, 2060.7)	1931.4 (1862.8, 2000.0)	1865.2 (1785.4, 1945.0)	1801.3 (1709.0, 1893.6)	1739.7 (1634.6, 1844.8)
	≥75	−1.6 (−2.8, −0.3)	6842.6 (6666.4, 7018.8)	6735.6 (6551.4, 6919.8)	6629.6 (6420.3, 6838.9)	6525.3 (6280.5, 6770.1)	6422.8 (6136.8, 6708.8)	6322.1 (5992.2, 6652.0)
Total^e^		···	1165.4 (1153.1, 1177.7)	1144.1 (1131.2, 1157.0)	1123.2 (1108.5, 1137.9)	1102.7 (1085.6, 1119.8)	1082.6 (1062.6, 1102.6)	1063.1 (1040.2, 1086.0)

APC indicates annual percent change.

a Based on the log‐linear models that were fitted to the 2006–2011 sex‐ and age‐specific rates and simulated 1000 times; used for stable baselines that are held constant during 2012–2016.

b Determined by subtracting the product of the preceding year's simulated rate multiplied by the 2006–2011 APC; used for the previous trend baselines during 2012–2016.

c APC significantly less compared with men aged 18 to 44 years (*P*=0.033).

d APC significantly greater compared with women aged 18 to 44 years (*P*=0.002).

e Rates are standardized by age to the 2010 US Census Population distribution among adults aged ≥18 years.

### Event Rates by Age and Sex

Event rates increased considerably with age (Figure 2). Relative rate differences by sex were most striking among the younger age groups. For example, in 2013, the event rate among men aged 45 to 64 years was 64.7% higher than the rate among like‐aged women. However, in the oldest age group (aged ≥75 years), the rate among men was only 5.9% higher than the rate among women. Women aged ≥75 years were the largest contributors to the event totals across the 8 sex/age groups in 2013, accounting for 26.5% of the events (Table S2).

### Event Rates by Type and Condition

Of the 2 829 161 (95% CI, 2 779 159, 2 879 163) events that occurred in 2013, 74.2% were hospitalizations (2 099 874 [95% CI, 2 053 475, 2 146 273]; Figure 1; Table S3); mortality contributed 13.6% of the events (386 073 [95% CI, 384 856, 387 290]) and ED encounters 12.1% of events (343 214 [95% CI, 324 621, 361 807]). The “other CVD conditions” category had the largest contribution to the overall event totals (47.9% of events; N=1 274 648 [95% CI, 1 263 527, 1 285 769]), with the largest contributor being heart failure–related hospitalizations (N=831 645 [95% CI, 822 075, 841 215]). AMI‐ and stroke‐related hospitalizations accounted for 45.8% (N=961 925 [95% CI, 952 127, 971 723]) of the hospitalization events, had the largest contribution (63.6%) to the death event totals (N=245 456 [95% CI, 244 485, 246 427]), and contributed 42.7% (N=1 207 381 [95% CI, 1 197 535, 1 217 227]) to the overall event count in 2013. Comparing the overall age‐standardized event rates in 2006 to 2013, by event category, the greatest relative overall reduction in rates occurred for the symptomatic precursor conditions (24.1% relative reduction); the smallest overall reduction occurred for stroke (0.3% relative reduction; Table 1). Moreover, despite considerable improvement in AMI event rates from 2006 to 2010, there was minimal improvement thereafter. Similarly, there was a slight increase in heart failure event rates in 2013 after experiencing declines during 2006 to 2012, which was driven by increases in the overall age‐standardized heart failure ED and mortality rates (increased from 63.8 and 29.5 per 100 000, in 2012, to 69.4 and 31.1 per 100 000, in 2013, respectively) and minimal change in the hospitalization rate (334.8 per 100 000, in 2012, compared with 333.7 per 100 000, in 2013; data not shown).

### Expected Event Rates and Events Prevented Estimates Using Both Baseline Strategies

The age‐standardized modeled 2011 event rate (per 100 000) for all adults aged ≥18 years was 1165.4 (95% CI, 1153.1, 1177.7), with the sex‐ and age‐specific event rates, which serve as the stable baselines, ranging from 106.3 (95% CI, 104.1, 108.5) among women aged 18 to 44 years to 7031.0 (95% CI, 6823.6, 7238.4) among men aged ≥75 years (Table 2). Using the trend baseline strategy and the simulation methodology, the overall age‐standardized event rate (per 100 000) is projected to be 1063.1 (95% CI, 1040.2, 1086.0) by 2016, with sex‐ and age‐specific rates ranging from 98.8 (95% CI, 94.7, 102.9) among women aged 18 to 44 years to 6439.8 (95% CI, 6068.8, 6810.8) among men aged ≥75 years. Overall, we estimate that 13.99 (trend baselines) to 14.81 (stable baselines) million events will occur during 2012–2016 without additional action, equating to an ≈820 000 event difference between the 2 baseline strategies over the 5 years.

**Figure 3:**
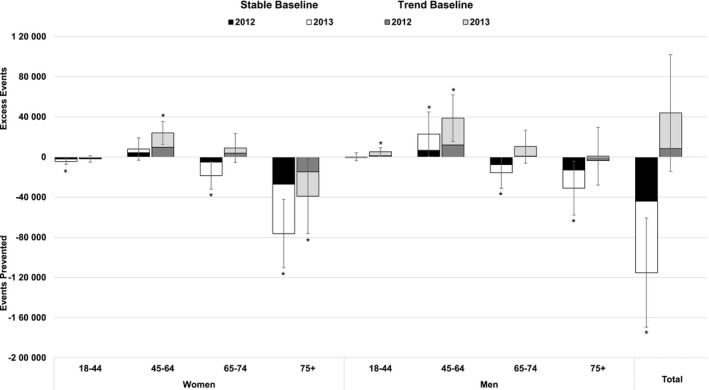
Estimated number of mutually exclusive Million Hearts events prevented in 2012 and 2013, by baseline strategy, sex, and age group. Determined by combining the observed event totals from the 2012 and 2013 and subtracting the expected number of events calculated by multiplying the 2011 modeled sex‐ and age‐specific rates (stable baselines) or by applying the annual percent change to the modeled 2011 rate for each sex‐ and age‐specific group to generate expected 2012 and 2013 rates (trend baselines) and multiplying those rates by the US Census mid‐year population estimates for 2012 and 2013, respectively. During 2012–2013, 115 210 (95% CI, 60 858, 169 562) events were prevented using the stable baseline; an excess of 43 934 (95% CI, −14 264, 102 132) events occurred using the trend baseline. Bars denote the 95% confidence intervals. Values denoted with an asterisk (“*”) are statistically significantly different than 0 (*z*‐score ≥|1.96|). Table S2 includes the sex‐ and age‐specific values for each year and for 2012 and 2013 combined.

Using the stable baseline strategy and comparing the expected versus observed sex‐ and age‐specific data, an estimated 44 012 (95% CI, 5723, 82 301) and 71 198 (95% CI, 32 622, 109 774) fewer events occurred than were expected in 2012 and 2013, respectively, for a total of 115 210 (95% CI, 60 858, 169 562) events prevented during the first 2 years of the initiative (statistically significantly different than 0; *P*<0.001; (Figure 3; Table S2). In comparison, if the past trends experienced during 2006–2011 were continued and used as the baseline, 8380 (95% CI, −30 833, 47 593) and 35 554 (95% CI, −7450, 78 558) more events occurred than were expected in 2012 and 2013, respectively, for a total excess in events of 43 934 (95% CI, −14 264, 102 132; not statistically significantly different than 0; *P*=0.139). Women aged ≥75 years had the most events prevented using either baseline strategy during the combined years of 2012 and 2013; 76 242 (95% CI, 42 067, 110 417) using a stable baseline and 39 049 (95% CI, 1901, 76 197) using a baseline incorporating previous trends. During 2012–2013, men and women aged 45 to 64 years had 22 912 (95% CI, 855, 44 969) and 7975 (95% CI, −3281, 19 231; stable baseline) to 38 810 (95% CI, 15 567, 62 053) and 24 080 (95% CI, 12 604, 35 556; previous trend baseline) more events, respectively, than were expected.

## Discussion

During the first 2 years of Million Hearts (2012–2013), improvement in event rates resulted in an estimated 115 000 events being prevented compared with what would have occurred had 2011 rates remained stable. However, there was no statistically significant change in the trajectory of the rates to demonstrate improvement beyond what would have happened had the trends observed during 2006–2011 continued. The choice to use 2 parallel baseline strategies—stable and trend baselines—to estimate the number of events prevented was made because the United States has to overcome several barriers, including an increasing prevalence of diabetes mellitus^33^ and obesity^34^ and an aging population,^9^ to continue making gains in event prevention. The effect these risk factors have on future event rates if they go unimpeded is uncertain; therefore, use of both strategies was considered a reasonable approach. We would consider attainment of the events prevented goal using either strategy a success. However, achieving the goal using the previous trends strategy would be ideal, given that an additional 820 000 events would be prevented if it was met using this strategy compared with only meeting it using the stable baseline strategy.

Although the events prevented during this period cannot be directly attributed to any single intervention or initiative, including Million Hearts, tracking these values is important because it demonstrates the collective progress the United States is making in reducing the burden of CVD and reveals event trends for specific conditions and among various populations. For example, during 2012–2013, stroke rates remained stable as they had since 2006. AMI rates, which decreased considerably up to 2010, demonstrated no improvement from 2010 to 2013, and heart failure rates, which decreased considerably up to 2012, increased slightly in 2013. The majority of the improvement in overall event rates that did occur during 2012–2013 happened among those aged ≥65 years, especially among women aged ≥75 years. Continued gradual improvement in event rates, in particular among this age group, was likely driven by increased use of cholesterol‐lowering medications,^35^ including 3‐hydroxy‐3‐methyl‐glutaryl‐coenzyme A reductase inhibitors (ie, statins), and moderate increases in blood pressure control.^36^ Continued improvement in CVD risk factors among this group will likely lead to the largest number of events prevented during the current initiative, given that they have the most events to prevent. In contrast, little to no improvement in event rates occurred among adults aged 45 to 64 years, with rates actually increasing among men aged 45 to 64. Although smoking prevalence continued to decrease among this age group,^37^ they remained at greater risk for being unaware or untreated for conditions like hypertension and hypercholesterolemia and less likely to be using aspirin for secondary CVD prevention compared with older adults.^19^, ^38^, ^39^ Moreover, this age cohort was born around 1960 and likely has been the generation most affected by the rise of the obesity epidemic.^40^ During their lifetime, the prevalence of obesity has more than doubled among adults,^40^ possibly placing them at increased risk for developing CVD resulting from overweight status and its associated risk factors compared with previous generations. Therefore, additional focus on preventing or delaying the development of CVD risk factors,^41^ identifying and treating the ABCS, implementing effective weight management interventions, and calculating and managing lifetime CVD risk,^42^ instead of just acute risk, is likely required to decrease event rates among this age group now and better position the United States for improved CVD outcomes in future decades.

There are potential limitations to this study. One potential limitation is that the event definition is based on mutually exclusive events, not the number of unique persons affected. Although this methodology tries to achieve mutual exclusivity among event totals, there is potential for survivor bias and over‐ or undercounting events. Moreover, changes in health‐ care utilization practices (eg, use of urgent care services instead of ED services) might affect rates rather than changes in disease burden. However, we have attempted to account for this in several ways. First, we excluded all hospitalizations characterized as elective, because how these cases are managed (eg, within acute care hospitals or in outpatient surgical centers) might change over time. Second, by including ED event data, we address potential changes in practice patterns where cases that were previously hospitalized are now managed in the ED setting, including through observation. Finally, the definition relies on use of administrative data; thus, reductions in the number of “silent” events, which account for upward of 20% of all AMIs and strokes^1^, ^43^, ^44^ and are likely affected by the focused attention on the leading risk factors, are not captured in monitoring systems and cannot be accounted for. Despite these limitations, the current definition uses the best available data sources to understand the number of unique Million Hearts–related fatal and nonfatal events prevented at a national level and across all adult age groups and health insurance coverage types, including people without health insurance. The use of this novel methodology helps address the need for improved comprehensive national cardiovascular event surveillance.^23^

The NIS was selected to describe the hospitalization burden because it is a robust data set that can be weighted to produce national estimates. While the NIS was redesigned in 2012, use of the Agency for Healthcare Research and Quality's trend weights alleviates differences attributed to sampling rather than changes in utilization during the observation period and the new design is expected to provide more‐precise results.^26^ The benefits of using the NEDS for ED surveillance has been described previously.^45^ Although the NEDS does not contain all of the same hospitals as the NIS sample, it is structured similarly; therefore, it better lends itself to the application of the exclusion criteria used to determine mutually exclusive events compared with data sets that are structured differently from the NIS. The biggest potential limitations with use of the NEDS are that the ability to assess trends is somewhat limited because it only provides data since 2006, and, depending on the year, the weighted national estimates are based on discharge data from only 24 to 31 states. However, NEDS allows considerable flexibility in analyses because of its large sample size.

The NVSS was selected to describe the death burden because it is the national standard for describing national death rates and has been used for tracking purposes in scientific publications^1^ and by various national and international projects, including the Healthy People initiative. A potential limitation of using the NVSS, as well as the Healthcare Cost and Utilization Project databases, is that they are nonvalidated administrative databases. Use of administrative claims data for surveillance can be affected by reporting or coding changes and has been found to sometimes overestimate cardiovascular event rates, especially deaths.^46^ However, the analysis of these data to assess cardiovascular event rate trends are recommended for national surveillance and, when combined across communities, appears valid given that the overestimation likely will not vary greatly at the macro level and over this short period.^46^, ^47^, ^48^

Because of the current lag time in data release for each of the data assets used for event surveillance, methodologies and strategies are being explored to forecast and/or calculate more‐timely preliminary estimates. In addition, the United States switched from the International Classification of Diseases, Ninth Revision, Clinical Modification–based claims system to the International Classification of Diseases, Tenth Revision, Clinical Modification–based system in October 2015. Crosswalks from the old to the new coding systems have been developed (Table S4); assessment of the effects of applying these new definitions on event totals is underway.

The United States will experience an ≈6.8% reduction in the total number of events that would have occurred during 2012–2016 by meeting the 1 million events prevented goal using the stable baseline strategy. Whereas only 12% of this goal was achieved in the first 2 years of the initiative through the prevention of an estimated 115 000 events, we expect that the implementation, and therefore the effects, of Million Hearts’ focused strategies on overall event totals will increase over time. However, the recent flattening observed in the overall event rate trend is concerning and supports intensifying national action in implementing community‐focused policy and systems changes and evidence‐based clinical treatment strategies to meet the aggressive, but achievable, targets of Million Hearts.^19^ In particular, the increase in event rates observed among those aged 45 to 64 years should serve as a warning that we need to better address the cardiovascular health of this cohort or a reversal in event rate trends may occur. Use of the novel methodology described in this study will allow for Million Hearts and its partners to understand the progress they are making toward their goal of preventing 1 million AMIs, strokes, and other related cardiovascular events through 2016 and beyond.

## Sources of Funding

The Centers for Disease Control and Prevention supported this study. No external funding was used. The findings and conclusions in this report are those of the authors and do not necessarily represent the official position of the Centers for Disease Control and Prevention or the Agency for Healthcare Research and Quality.

## Disclosures

None.

## Supporting information


**Data S1.** Supplemental methods.
**Table S1.** Million Hearts Case Definition Codes by Event Category and Type
**Table S2.** Number of Expected, Observed, and Estimated Prevented Million Hearts Events, by Baseline Strategy, in 2012 (A), 2013 (B), and 2012–2013 Combined (C), by Sex and Age Group
**Table S3.** Event Counts and Age‐Standardized Rates* by Event Category and Event Type and Data Source, 2013
**Table S4.** Post ICD‐10‐CM Transition Million Hearts Case Definition Codes by Event Category and Type
**Figure S1.** Detailed Million Hearts mutually exclusive event calculation methodology applied to the 2013 NEDS, NIS, and NVSS data.
**Figure S2.** Example of how the stable and previous trend baselines were established for each sex and age group, men aged 65 to 74 years.

## References

[1] Mozaffarian D , Benjamin EJ , Go AS , Arnett DK , Blaha MJ , Cushman M , Das SR , de Ferranti S , Després JP , Fullerton HJ , Howard VJ , Huffman MD , Isasi CR , Jiménez MC , Judd SE , Kissela BM , Lichtman JH , Lisabeth LD , Liu S , Mackey RH , Magid DJ , McGuire DK , Mohler ER III , Moy CS , Muntner P , Mussolino ME , Nasir K , Neumar RW , Nichol G , Palaniappan L , Pandey DK , Reeves MJ , Rodriguez CJ , Rosamond W , Sorlie PD , Stein J , Towfighi A , Turan TN , Virani SS , Woo D , Yeh RW , Turner MB ; American Heart Association Statistics Committee; Stroke Statistics Subcommittee . Heart disease and stroke statistics—2016 update: a report from the American Heart Association. Circulation. 2016;133:e38–e360. 26673558 10.1161/CIR.0000000000000350

[2] Centers for Disease Control and Prevention . Prevalence and most common causes of disability among adults—United States, 2005. MMWR Morb Mortal Wkly Rep. 2009;58:421–426. 19407734

[3] Murphy SL , Kochanek KD , Xu JQ , Arias E . Mortality in the United States, 2014. NCHS data brief, no 229. Hyattsville, MD: National Center for Health Statistics; 2015. 26727391

[4] Centers for Disease Control and Prevention . Vital signs: avoidable deaths from heart disease, stroke, and hypertensive disease—United States, 2001–2010. MMWR Morb Mortal Wkly Rep. 2013;62:721–727. 24005227 PMC4585625

[5] Nyweide DJ , Anthony DL , Bynum JP , Strawderman RL , Weeks WB , Casalino LP , Fisher ES . Continuity of care and the risk of preventable hospitalization in older adults. JAMA Intern Med. 2013;173:1879–1885. 24043127 10.1001/jamainternmed.2013.10059 PMC3877937

[6] Moy E , Chang E , Barrett M . Potentially preventable hospitalizations—United States, 2001–2009. MMWR Surveill Summ. 2013;62:139–143. 24264504

[7] Agency for Health Care Research and Quality . Guide to prevention quality indicators: hospital admission for ambulatory care sensitive conditions. Publication No. 02‐R0203. Rockville, MD; 2007 Available at: http://www.qualityindicators.ahrq.gov/Downloads/Modules/PQI/V31/pqi_guide_v31.pdf. Accessed September 26, 2016.

[8] Krumholz HM , Normand SL , Wang Y . Trends in hospitalizations and outcomes for acute cardiovascular disease and stroke: 1999–2011. Circulation. 2014;130:966–975. 25135276 10.1161/CIRCULATIONAHA.113.007787 PMC4171056

[9] US Census Bureau, Population Division . Projected population by single year of age, sex, race, and Hispanic origin for the United States: 2014 to 2060. 2014 Available at: https://www.census.gov/population/projections/data/national/2014.html. Accessed September 26, 2016.

[10] Heidenreich PA , Trogdon JG , Khavjou OA , Butler J , Dracup K , Ezekowitz MD , Finkelstein EA , Hong Y , Johnston SC , Khera A , Lloyd‐Jones DM , Nelson SA , Nichol G , Orenstein D , Wilson PW , Woo YJ ; American Heart Association Advocacy Coordinating Committee; Stroke Council; Council on Cardiovascular Radiology and Intervention; Council on Clinical Cardiology; Council on Epidemiology and Prevention; Council on Arteriosclerosis; Thrombosis and Vascular Biology; Council on Cardiopulmonary; Critical Care; Perioperative and Resuscitation; Council on Cardiovascular Nursing; Council on the Kidney in Cardiovascular Disease; Council on Cardiovascular Surgery and Anesthesia, and Interdisciplinary Council on Quality of Care and Outcomes Research . Forecasting the future of cardiovascular disease in the United States: a policy statement from the American Heart Association. Circulation. 2011;123:933–944. 21262990 10.1161/CIR.0b013e31820a55f5

[11] Odden MC , Coxson PG , Moran A , Lightwood JM , Goldman L , Bibbins‐Domingo K . The impact of the aging population on coronary heart disease in the United States. Am J Med. 2011;124:827–833. 21722862 10.1016/j.amjmed.2011.04.010 PMC3159777

[12] Pearson‐Stuttard J , Guzman‐Castillo M , Penalvo JL , Rehm CD , Afshin A , Danaei G , Kypridemos C , Gaziano T , Mozaffarian D , Capewell S , O'Flaherty M . Modeling future cardiovascular disease mortality in the United States: national trends and racial and ethnic disparities. Circulation. 2016;133:967–978. 26846769 10.1161/CIRCULATIONAHA.115.019904 PMC4783256

[13] Wilmot KA , O'Flaherty M , Capewell S , Ford ES , Vaccarino V . Coronary heart disease mortality declines in the United States from 1979 through 2011: evidence for stagnation in young adults, especially women. Circulation. 2015;132:997–1002. 26302759 10.1161/CIRCULATIONAHA.115.015293 PMC4828724

[14] Ford ES , Capewell S . Proportion of the decline in cardiovascular mortality disease due to prevention versus treatment: public health versus clinical care. Annu Rev Public Health. 2011;32:5–22. 21417752 10.1146/annurev-publhealth-031210-101211

[15] Farley TA , Dalal MA , Mostashari F , Frieden TR . Deaths preventable in the U.S. by improvements in use of clinical preventive services. Am J Prev Med. 2010;38:600–609. 20494236 10.1016/j.amepre.2010.02.016

[16] Towfighi A , Saver JL . Stroke declines from third to fourth leading cause of death in the United States: historical perspective and challenges ahead. Stroke. 2011;42:2351–2355. 21778445 10.1161/STROKEAHA.111.621904

[17] Frieden TR , Berwick DM . The “Million Hearts” initiative—preventing heart attacks and strokes. N Engl J Med. 2011;365:e27. 21913835 10.1056/NEJMp1110421

[18] Million Hearts: strategies to reduce the prevalence of leading cardiovascular disease risk factors—United States, 2011. MMWR Morb Mortal Wkly Rep. 2011;60:1248–1251. 21918495

[19] Ritchey MD , Wall HK , Gillespie C , George MG , Jamal A . Million Hearts: prevalence of leading cardiovascular disease risk factors—United States, 2005–2012. MMWR Morb Mortal Wkly Rep. 2014;63:462–467. 24871251 PMC5779465

[20] Bibbins‐Domingo K , Chertow GM , Coxson PG , Moran A , Lightwood JM , Pleatcher MJ , Goldman L . Projected effect of dietary salt reductions on future cardiovascular disease. N Engl J Med. 2010;362:590–599. 20089957 10.1056/NEJMoa0907355 PMC3066566

[21] Hunink MG , Goldman L , Tosteson AN , Mittleman MA , Goldman PA , Williams LW , Tsevat J , Weinstein MC . The recent decline in mortality from coronary heart disease, 1980–1990. The effect of secular trends in risk factors and treatment. JAMA. 1997;277:535–542. 9032159

[22] Antiplatelet Trialists’ Collaboration . Collaborative overview of randomised trials of antiplatelet therapy. I. Prevention of death, myocardial infarction, and stroke by prolonged antiplatelet therapy in various categories of patients. BMJ. 1994;308:81–106. [Erratum, BMJ 1994;308:1540.] 8298418 PMC2539220

[23] Goff DC Jr , Brass L , Braun LT , Croft JB , Flesch JD , Fowkes FG , Hong Y , Howard V , Huston S , Jencks SF , Luepker R , Manolio T , O'Donnell C , Robertson RM , Rosamond W , Rumsfeld J , Sidney S , Zheng ZJ ; American Heart Association Council on Epidemiology and Prevention; American Heart Association Council on Stroke; American Heart Association Council on Cardiovascular Nursing; American Heart Association Working Group on Quality of Care and Outcomes Research; American Heart Association Working Group on Atherosclerotic Peripheral Vascular Disease . Essential features of a surveillance system to support the prevention and management of heart disease and stroke: a scientific statement from the American Heart Association Councils on Epidemiology and Prevention, Stroke, and Cardiovascular Nursing and the Interdisciplinary Working Groups on Quality of Care and Outcomes Research and Atherosclerotic Peripheral Vascular Disease. Circulation. 2007;115:127–155. 17179025 10.1161/CIRCULATIONAHA.106.179904

[24] Agency for Healthcare Research and Quality . Healthcare Cost and Utilization Project Nationwide Emergency Department Sample database documentation. Rockville, MD; 2014 Available at: www.hcup-us.ahrq.gov/db/nation/neds/nedsdbdocumentation.jsp. Accessed September 26, 2016.

[25] Agency for Healthcare Research and Quality . Healthcare Cost and Utilization Project Nationwide Inpatient Sample database documentation. Rockville, MD; 2014 Available at: www.hcup-us.ahrq.gov/db/nation/nis/nisdbdocumentation.jsp. Accessed September 26, 2016.

[26] Agency for Healthcare Research and Quality . Nationwide inpatient sample redesign final report. Rockville, MD; 2014 Available at: http://www.hcup-us.ahrq.gov/db/nation/nis/reports/NISRedesignFinalReport040914.pdf. Accessed September 26, 2016.

[27] Agency for Healthcare Research and Quality . Trend weights for Healthcare Cost and Utilization Project Nationwide Inpatient Sample data. Rockville, MD; 2015 Available at: http://www.hcup-us.ahrq.gov/db/nation/nis/trendwghts.jsp. Accessed September 26, 2016.

[28] National Center for Health Statistics . Vital statistics of the United States: mortality, 1999 technical appendix. Hyattsville, MD; 1999 Available at: http://wonder.cdc.gov/wonder/sci_data/mort/mcmort/type_txt/mcmort05/techap99.pdf. Accessed September 26, 2016.

[29] National Center for Health Statistics . International classification of diseases, ninth revision, clinical modification (ICD‐9‐CM). Hyattsville, MD; 2013 Available at: http://www.cdc.gov/nchs/icd/icd9cm.htm. Accessed September 26, 2016.

[30] World Health Organization . International Statistical Classification of Diseases and Related Health Problems, 10th Revision (ICD–10). 2nd ed Geneva: Switzerland; 2004.

[31] United States Department of Health and Human Services, Centers for Disease Control and Prevention, National Center for Health Statistics (NCHS), Bridged‐Race Population Estimates, United States July 1st resident population by state, county, age, sex, bridged‐race, and Hispanic origin. Compiled from 1990‐1999 bridged‐race intercensal population estimates (released by NCHS on 7/26/2004); revised bridged‐race 2000‐2009 intercensal population estimates (released by NCHS on 10/26/2012); and bridged‐race Vintage 2015 (2010‐2015) postcensal population estimates (released by NCHS on 6/28/2016). Available at: http://wonder.cdc.gov/bridged-race-v2015.html. Accessed September 26, 2016.

[32] Clegg LX , Hankey BF , Tiwari R , Feuer EJ , Edwards BK . Estimating average annual percent change in trend analysis. Stat Med. 2009;28:3670–3682. 19856324 10.1002/sim.3733 PMC2843083

[33] Centers for Disease Control and Prevention . National Diabetes Fact Sheet: National Estimates and General Information on Diabetes and Prediabetes in the United States, 2011. Atlanta, GA: U.S. Department of Health and Human Services, Centers for Disease Control and Prevention, 2011.

[34] Ogden CL , Carroll MD , Kit BK , Flegal KM . Prevalence of obesity among adults: United States, 2011–2012. NCHS data brief, no 131. Hyattsville, MD: National Center for Health Statistics; 2013. 24152742

[35] Gu Q , Paulose‐Ram R , Burt VL , Kit BK . Prescription cholesterol‐lowering medication use in adults aged 40 and over: United States, 2003–2012. NCHS data brief, no 177. Hyattsville, MD: National Center for Health Statistics; 2014. 25536410

[36] Yoon SS , Fryar CD , Carroll MD . Hypertension prevalence and control among adults: United States, 2011–2014. NCHS data brief, no 220. Hyattsville, MD: National Center for Health Statistics; 2015. 26633197

[37] Centers for Disease Control and Prevention . Trends in current cigarette smoking among high school students and adults, United States, 1965–2014. Atlanta, GA; 2016 Available at: http://www.cdc.gov/tobacco/data_statistics/tables/trends/cig_smoking/. Accessed September 26, 2016.

[38] Centers for Disease Control and Prevention . Vital signs: awareness and treatment of uncontrolled hypertension among adults—United States, 2003–2010. MMWR Morb Mortal Wkly Rep. 2012;61:703–709. 22951452

[39] Centers for Disease Control and Prevention . Vital signs: prevalence, treatment, and control of high levels of low‐density lipoprotein cholesterol–United States, 1999–2002 and 2005–2008. MMWR Morb Mortal Wkly Rep. 2011;60:109–114. 21293326

[40] Ogden CL , Carroll MD . Prevalence of overweight, obesity, and extreme obesity among adults: united states, trends 1960–1962 through 2007–2008. NCHS Health E‐Stat. Hyattsville, MD: National Center for Health Statistics; 2010.

[41] Ahmad FS , Ning H , Rich JD , Yancy CW , Lloyd‐Jones DM , Wilkins JT . Hypertension, obesity, diabetes, and heart failure‐free survival: the Cardiovascular Disease Lifetime Risk Pooling Project. JACC Heart Fail. 2016;4:911–919. 27908389 10.1016/j.jchf.2016.08.001 PMC5582802

[42] Yang Q , Zhong Y , Ritchey M , Cobain M , Gillespie C , Merritt R , Hong Y , George MG , Bowman BA . Vital signs: predicted heart age and racial disparities in heart age among U.S. adults at the state level. MMWR Morb Mortal Wkly Rep. 2015;64:950–958. 26335037 10.15585/mmwr.mm6434a6

[43] Fanning JP , Wong AA , Fraser JF . The epidemiology of silent brain infarction: a systematic review of population‐based cohorts. BMC Med. 2014;12:119. 25012298 10.1186/s12916-014-0119-0 PMC4226994

[44] Kim HW , Klem I , Shah DJ , Wu E , Meyers SN , Parker MA , Crowley AL , Bonow RO , Judd RM , Kim RJ . Unrecognized non‐Q‐wave myocardial infarction: prevalence and prognostic significance in patients with suspected coronary disease. PLoS Med. 2009;6:e1000057. 19381280 10.1371/journal.pmed.1000057 PMC2661255

[45] Owens PL , Barrett ML , Gibson TB , Andrews RM , Weinick RM , Mutter RL . Emergency department care in the United States: a profile of national data sources. Ann Emerg Med. 2010;56:150–165. 20074834 10.1016/j.annemergmed.2009.11.022

[46] Coady SA , Sorlie PD , Cooper LS , Folsom AR , Rosamond WD , Conwill DE . Validation of death certificate diagnosis for coronary heart disease: the Atherosclerosis Risk in Communities Study. J Clin Epidemiol. 2001;54:40–50. 11165467 10.1016/s0895-4356(00)00272-9

[47] Institute of Medicine . A Nationwide Framework for Surveillance of Cardiovascular and Chronic Lung Diseases. Washington, DC: The National Academies Press; 2011. 22259816

[48] McCormick N , Lacaille D , Bhole V , Avina‐Zubieta JA . Validity of myocardial infarction diagnoses in administrative databases: a systematic review. PLoS One. 2014;9:e92286. 24682186 10.1371/journal.pone.0092286 PMC3969323

