# Supplementary material for: Million Hearts: Description of the National Surveillance and Modeling Methodology Used to Monitor the Number of Cardiovascular Events Prevented During 2012–2016
Source: J Am Heart Assoc. 2017 May 2;6(5):e006021. doi: 10.1161/JAHA.117.006021 (PMC5524118; doi:10.1161/JAHA.117.006021)
Supplement: Supplementary file 1 — Data S1. Supplemental methods. Table S1. Million Hearts Case Definition Codes by Event Category and Type Table S2. Number of Expected, Observed, and Estimated Prevented Million Hearts Events, by Baseline Strategy, in 2012 (A), 2013 (B), and 2012–2013 Combined (C), by Sex and Age Group Table S3. Event Counts and Age‐Standardized Rates* by Event Category and Event Type and Data Source, 2013 Table S4. Post ICD‐10‐CM Transition Million Hearts Case Definition Codes by Event Category and Type Figure S1. Detailed Million Hearts mutually exclusive event calculation methodology applied to the 2013 NEDS, NIS, and NVSS data. Figure S2. Example of how the stable and previous trend baselines were established for each sex and age group, men aged 65 to 74 years. [file JAH3-6-e006021-s001.pdf]

SUPPLEMENTAL MATERIAL

Data S1.

SUPPLEMENTAL METHODS

Decision Points When Developing the Million Hearts Event Definition

Multiple decision points were discussed during the development of the Million Hearts events case definition. Inclusion of each *International Classification of Disease*, *Ninth Revision, Clinical Modification* (ICD-9-CM) or *International Classification of Diseases, Tenth Revision* (ICD-10) component was driven by: 1) administrative codes that can be adequately used to describe the condition of interest; 2) codes that typically signal events that are acute in nature; 3) events that are most likely not elective hospital encounters; 4) alignment with current HHS agencies' definitions; and, 5) events that could be expected to be prevented by current or planned Million Hearts efforts. Many of the other decision points are discussed here:

1) Should the "symptomatic precursor conditions" be included in the Million Hearts events definition and at what level should they be tracked? Yes, stable angina, other acute ischemic heart disease (e.g., unstable angina), transient ischemic attacks, and hypertension should be included in the definition, because they are conditions that: can cause overt symptoms that would potentially lead patients to seek emergency care; are typically non-fatal, especially when treated early; and are usually highly preventable.[1,2] The first three event types remain in the definition across all settings, because they are acute cardiovascular conditions that are often managed in emergency departments (EDs) and during hospitalizations and are highly preventable by Million Hearts activities. Deaths attributable to these conditions are rare, but they were still included in the definition. Hypertension is discussed further below.

2) Should hypertension be included and at what level? Overall, Million Hearts activities should positively affect hypertension-related ED, hospitalization, and death events via its blood pressure control activities. However, hypertension-related ED encounters were excluded from the definition because ED services can often be used for hypertension management and not necessarily to treat acute events. With improvements in health insurance coverage through the enactment of the Patient Protection and Affordable Care Act, greater access to primary care services may alter how hypertension is being

managed (e.g., clinic-based versus ED-based) for certain groups and changes in event rates may be more indicative of changes in patient access rather than burden.[3-5] Hypertension-related hospitalizations and deaths are included in the definition, because current Million Hearts efforts to improve blood pressure control should prevent these acute events from occurring.[6]

3) Should heart failure be included in the definition? Or, should only acute heart failure be included? Heart failure events can be prevented by Million Hearts activities. Specifically, improvements in medication adherence[7-9], a decreased incidence of heart attacks[10], and improved hypertension control[10] should prevent heart failure-related ED encounters, hospitalizations, and deaths. Originally, we attempted to only include acute heart failure events in the case definition. However, despite the availability of specific codes to identify acute heart failure ED and hospitalization events (ICD-9-CM: 428.21, 428.23, 428.31, 428.33, 428.41, and 428.43) it was found that changes in coding practices during the observation period demonstrated that these codes might not accurately describe the acuity of the event (data not shown). Therefore, all heart failure event codes (ICD-9-CM: 428.XX) were included in definition.

4) Should acute myocardial infarctions, strokes, and other highly acute ED events be included? A considerable number of higher acuity events (e.g., AMIs and strokes) occurred in the ED. While the vast majority of patients experiencing these events were later hospitalized or died in the ED or hospital and, therefore, were theoretically captured in one of the other two surveillance systems, multiple cases were treated and then released from the hospital. Because of the acuity of these event types, it is expected all patients should have been admitted, transferred, or died. Therefore, we excluded all of the higher acuity ED cases, because they should be captured in the other data sets; those who were not likely had advance directives, were coded incorrectly, or left against medical advice.

5) Should all stroke deaths be included? We included hospitalization and mortality codes that were acute and directly related to ischemic or hemorrhagic strokes, because it was determined that Million Hearts activities can directly impact those conditions. While there is a link between Million Hearts activities (e.g., improving blood pressure control) and decreasing the burden of other types of strokes (e.g., non-traumatic subarachnoid hemorrhages[11]), those effects are more distal and may not greatly impact

hospitalizations; therefore, strokes other than ischemic or hemorrhagic strokes were only included in the mortality definition.

Calculation of Standard Errors for the Mortality Data and Combination of Estimates Across Datasets

Mortality data represent complete counts of all deaths, therefore, they are not subject to sampling error. However, these data are subject to non-sampling error resulting from potential errors in the death reporting process, therefore, we calculated and report the estimates and standard errors for the mortality counts per the methodology used by the National Center for Health Statistics.[12] The mortality estimates and standard errors were then combined with the weighted estimates and standard deviations derived from the Nationwide Emergency Department Sample and Nationwide/National Inpatient Sample analyses (using the SAS PROC SURVEYFREQ procedure) for the ED visits and hospitalizations, respectively, to report the combined overall event counts and standard deviations. The choice to use the mortality estimates' standard errors for combination, instead of converting the standard errors to standard deviations, was made for two reasons: 1) mortality estimates are not subject to sampling error, therefore, it was determined inappropriate to attempt to calculate standard deviations; and 2) converting the standard errors to standard deviations using the equation: standard deviation = standard error * sqrt (population n) would greatly overestimate error in the mortality estimates.

Additional Information about Log-linear Model Development and Use to Establish Stable and Previous Trend Baselines and Interpret Progress

The sex- and age-specific rates from 2006–2011 were each fit with a log-linear regression model. The log-transformed beta obtained from each model was then applied within this equation [exp(beta)-1] to describe the annual percent change (APC) in the rates. Because we only had access to 6 years of historical data there wasn't enough available data to appropriately assess for statistically significant breaks in the rate trend using Joinpoint methods or piecewise linear Poisson models. However, during our preliminary analyses we assessed the rates in Joinpoint,[13,14] and no significant trend breaks were identified (P>.05).

Here is how we established the two baselines (stable and previous trends) for each sex- and age-specific group, using men aged 65–74 years as an example (Figure S2). To establish their stable baseline rate, we held their modeled 2011 rate (2827.4 per 100000; Table 2) constant during 2012–2016. To establish their previous trend baseline, we applied their APC (-2.7%; Table 2) to the modeled 2011 rate to get their estimated 2012 rate (2752.1 per 100000). This was then repeated for each year through 2016. Each baseline strategy was simulated 1000 times using the sex- and age-specific modeled 2011 rates and, for the previous trends baseline, the sex- and age-specific APCs with their associated standard errors. The means and associated standard deviations of these simulated rates, in combination with US Census population estimates, were then used to compare to the events observed during 2012 and 2013. Overall, we consider it a success if the observed rate for each/all of the sex- and age-specific groups falls within one of the two green sections (Figure S2). Ideally, it would fall in the darkest green section meaning the event rate decrease was accelerated above and beyond what had occurred previously. However, in this example, the observed rates falls in the light green section, meaning that events were prevented compared with what would have occurred had the 2011 rates remained stable.

Table S1. Million Hearts case definition codes by event category and type

Event Category or Type	Emergency Department Primary ICD-9-CM Codes*	Acute Care Hospitalization Primary ICD-9-CM Codes*	Underlying Cause of Death ICD-10 Codes*
**Acute myocardial infarction**	—	410.X0, 410.X1	I21
**Stroke**			
Acute ischemic cerebral infarction	—	433.X1, 434.X1	I63
Acute hemorrhagic stroke	—	430, 431	I60, I61
Acute, but ill-defined, cerebrovascular disease	—	436	— †
Other cerebrovascular disease deaths	—	—	I62, I64, I67, I69
**Symptomatic precursor conditions**			
Other acute and subacute ischemic heart disease	411	411	I20.0‡, I24
Stable angina pectoris	413	413	I20.1, I20.8, I20.9
Transient ischemic attack	435	435	G45.0, G45.1, G45.2, G45.8, G45.9
**Other cardiovascular disease conditions**			
Heart failure	[402.X1, 404.X1]§, 428	[402.X1, 404.X1]§, 428	[I11.0, I13.0]‖, I50
Abdominal aortic aneurysms	—	441.02, 441.3#	I71.3, I71.4**
Atheroembolism	—	445	— ††

Atherosclerosis and peripheral artery disease	—	—	[I70, I73.9[‡‡]][§§]
Hypertension without heart failure	—	[401, 402.X0, 403.X0, 404.X0][§]	[I10, I11.9, I12.9, I13.9][‖]
Cardiac arrest	—	427.5[‖‖]	I46[‖‖]

Abbreviations: ICD-9-CM, International Classification of Diseases, 9th Revision, Clinical Modification codes; ICD-10, International Classification of Diseases, 10th Revision

* Excludes all cases involving women with any pregnancy-related ICD-9-CM codes (630–677, V22, V23, or V28) or contributing cause of death ICD-10 codes (O00–O99).

† ICD-9-CM code 436 does not accurately map to an ICD-10 death code (closest code: I64, which is included as “Other cerebrovascular disease deaths”).

‡ “Unstable angina” (i.e. intermediate coronary syndrome, ICD-9-CM 411.1) does not map to an I24 ICD-10 code, but to I20.0.

§ Excludes people with a chronic kidney disease, stage 5, or end-stage renal disease ICD-9-CM code of 585.5, 585.6, V42.0, V45.1, or V56.

|| Excludes people with a chronic kidney disease, stage 5 ICD-10 contributing cause of death code of N18.5.

Includes only acute abdominal aortic dissection and rupture (not asymptomatic or elective admissions for surgery).

** “Abdominal aortic aneurysm without mention of rupture” is included because—although not explicitly mentioned—deaths from abdominal aortic aneurysm almost always occur as the result of rupture.

†† ICD-10 mortality codes for embolism/thrombosis (I74) do not specify the cause of the embolism/thrombosis like ICD-9-CM codes do, therefore they were excluded.

‡‡ “Peripheral vascular disease, unspecified” is included (I73.9), because it best represents deaths attributable to “peripheral artery disease.”

§§ Only deaths attributable to atherosclerosis or peripheral artery disease were included because Million Hearts activities will directly impact mortality related to these types of cases, but not necessarily emergency department visits or hospitalizations.

|||| Cardiac events were only included if they had a secondary diagnosis/contributing cause of death described using a Million Hearts ICD-9-CM diagnosis or ICD-10 death code.

Table S2. Number of expected, observed, and estimated prevented Million Hearts events, by baseline strategy, in 2012 (a), 2013 (b), and 2012–2013 combined (c), by sex and age group

a. Year: 2012

	Expected Events: Stable Baseline	Expected Events: Trend Baseline	Observed Events	Events Prevented: Stable Baseline				Events Prevented: Trend Baseline			
**Sex and Age Group (y)**	**Number**	**Number**	**Number**	**Number**	**Lower 95% CI**	**Upper 95% CI**	**z-score**	**Number**	**Lower 95% CI**	**Upper 95% CI**	**z-score**
Men, 18-44	88,048	86,190	87,563	-485	-3321	2351	0.34	1373	-1484	4230	-0.94
Men, 45-64	478,861	473,561	485,544	6683	-8960	22,326	-0.84	11,983	-3910	27,876	-1.48
Men, 65-74	317,035	308,599	309,505	-7530	-18,384	3324	1.36	906	-10,088	11,900	-0.16
Men, 75+	535,507	526,226	522,562	-12,945	-31,698	5808	1.35	-3664	-22,890	15,562	0.37
Women, 18-44	60,287	59,416	58,261	-2026	-4252	200	1.78	-1155	-3418	1108	1.00
Women, 45-64	312,351	306,978	316,737	4386	-3636	12,408	-1.07	9759	1686	17,832	-2.37
Women, 65-74	264,853	255,921	259,806	-5047	-14,556	4462	1.04	3885	-5788	13,558	-0.79
Women, 75+	789,325	776,984	762,277	-27,048	-51,215	-2881	2.19	-14,707	-39,659	10,245	1.16
**Total**	**2,846,267**	**2,793,875**	**2,802,255**	**-44,012**	**-82,301**	**-5723**	**2.25**	**8380**	**-30,833**	**47,593**	**-0.42**

b. Year: 2013

Sex and Age Group (y)	Expected Events: Stable Baseline	Expected Events: Trend Baseline	Observed Events	Events Prevented: Stable Baseline				Events Prevented: Trend Baseline			
	Number	Number	Number	Number	Lower 95% CI	Upper 95% CI	z-score	Number	Lower 95% CI	Upper 95% CI	z-score
Men, 18-44	88,622	84,902	88,827	205	-2,576	2986	-0.14	3925	1051	6799	-2.68
Men, 45-64	480,354	469,756	496,583	16,229	679	31,779	-2.05	26,827	9866	43,788	-3.10
Men, 65-74	333,444	315,821	325,284	-8160	-19,403	3083	1.42	9463	-2793	21,719	-1.51
Men, 75+	547,911	529,009	529,847	-18,064	-37,218	1090	1.85	838	-20,560	22,236	-0.08
Women, 18-44	60,601	58,857	58,192	-2409	-4555	-263	2.20	-665	-2960	1630	0.57
Women, 45-64	313,290	302,558	316,879	3589	-4307	11,485	-0.89	14,321	6164	22,478	-3.44
Women, 65-74	277,915	259,334	264,521	-13,394	-23,115	-3673	2.70	5187	-5589	15,963	-0.94
Women, 75+	798,222	773,370	749,028	-49,194	-73,357	-25,031	3.99	-24,342	-51,863	3179	1.73
**Total**	**2,900,359**	**2,793,607**	**2,829,161**	**-71,198**	**-109,774**	**-32,622**	**3.62**	**35,554**	**-7450**	**78,558**	**-1.62**

c. Years: 2012 and 2013 combined

	Expected Events: Stable Baseline	Expected Events: Trend Baseline	Observed Events	Events Prevented: Stable Baseline				Events Prevented: Trend Baseline			
**Sex and Age Group (y)**	**Number**	**Number**	**Number**	**Number**	**Lower 95% CI**	**Upper 95% CI**	**z-score**	**Number**	**Lower 95% CI**	**Upper 95% CI**	**z-score**
Men, 18-44	176,670	171,092	176,390	-280	-4252	3692	0.14	5298	1246	9350	-2.56
Men, 45-64	959,215	943,317	982,127	22,912	855	44,969	-2.04	38,810	15,567	62,053	-3.27
Men, 65-74	650,479	624,420	634,789	-15,690	-31,318	-62	1.97	10,369	-6095	26,833	-1.23
Men, 75+	1,083,418	1,055,235	1,052,409	-31,009	-57,815	-4203	2.27	-2826	-31,593	25,941	0.19
Women, 18-44	120,888	118,273	116,453	-4435	-7527	-1343	2.81	-1820	-5043	1403	1.11
Women, 45-64	625,641	609,536	633,616	7975	-3281	19,231	-1.39	24,080	12,604	35,556	-4.11
Women, 65-74	542,768	515,255	524,327	-18,441	-32,039	-4843	2.66	9072	-5409	23,553	-1.23
Women, 75+	1,587,547	1,550,354	1,511,305	-76,242	-110,417	-42,067	4.37	-39,049	-76,197	-1901	2.06
**Total**	**5,746,626**	**5,587,482**	**5,631,416**	**-115,210**	**-169,562**	**-60,858**	**4.15**	**43,934**	**-14,264**	**102,132**	**-1.48**

Abbreviation: CI, confidence interval

Table S3. Event counts and age-standardized rates* by event category and event type and data source, 2013

Event Category or Type	ED visits		Hospitalizations		Deaths		Total events	
	Events n (95% CI)	Rate (95% CI)	Events n (95% CI)	Rate (95% CI)	Events n (95% CI)†	Rate (95% CI)†	Events n (95% CI)	Rate (95% CI)
**Total**	**343214 (324621, 361807)**	**138.1 (133.3, 142.9)**	**2099874 (2053475, 2146273)**	**844.1 (832.4, 855.8)**	**386073 (384855, 387291)**	**155.4 (154.9, 155.8)**	**2829161 (2779160, 2879162)**	**1137.6 (1125, 1150.2)**
**Acute myocardial infarction**	—	—	**489715 (481666, 497764)**	**197.1 (193.8, 200.3)**	**116749 (116079, 117419)**	**46.8 (46.6, 47.1)**	**606464 (598388, 614540)**	**243.9 (240.6, 247.2)**
**Stroke**	—	—	**472210 (466622, 477798)**	**189.6 (187.3, 191.8)**	**128707 (128004, 129410)**	**51.8 (51.5, 52.0)**	**600917 (595285, 606549)**	**241.3 (239.1, 243.6)**
Acute ischemic cerebral infarction	—	—	410830 (405527, 416133)	164.8 (162.6, 166.9)	6694 (6534, 6854)	2.7 (2.6, 2.8)	417524 (412219, 422829)	167.5 (165.3, 169.6)
Acute hemorrhagic stroke	—	—	60910 (59151, 62669)	24.6 (23.9, 25.3)	21285 (20999, 21571)	8.6 (8.5, 8.7)	82195 (80413, 83977)	33.2 (32.5, 33.9)
Acute, but ill-defined, cerebrovascular disease	—	—	470 (370, 570)	0.2 (0.1, 0.2)	—	—	470 (370, 570)	0.2 (0.1, 0.2)
Other cerebrovascular disease deaths	—	—	—	—	100728 (100106, 101350)	40.5 (40.2, 40.7)	100728 (100106, 101350)	40.5 (40.2, 40.7)
**Symptomatic precursor conditions**	**170571 (166586, 174555)**	**68.7 (67.1, 70.3)**	**172345 (169815, 174875)**	**69.3 (68.3, 70.3)**	**4216 (4089, 4343)**	**1.7 (1.6, 1.7)**	**347132 (342410, 351854)**	**139.7 (137.8, 141.6)**

Other acute and subacute ischemic heart disease	26062<br>(24694, 27430)	10.6<br>(10.0, 11.1)	18710<br>(17996, 19424)	7.6<br>(7.3, 7.9)	3954<br>(3831, 4077)	1.6<br>(1.5, 1.6)	48726<br>(47178, 50274)	19.7<br>(19.1, 20.3)
Stable angina pectoris	28073<br>(26847, 29299)	11.4<br>(10.9, 11.9)	9840<br>(9318, 10362)	4.0<br>(3.8, 4.2)	157<br>(132, 182)	0.1<br>(0.1, 0.1)	38070<br>(36737, 39403)	15.4<br>(14.9, 16.0)
Transient ischemic attack	116436<br>(112900, 119972)	46.8<br>(45.3, 48.2)	143795<br>(141424, 146166)	57.7<br>(56.8, 58.7)	105<br>(85, 125)	<0.1<br>(<0.1, 0.1)	260336<br>(256079, 264593)	104.5<br>(102.8, 106.2)
**Other cardiovascular disease conditions**	**172642<br>(167469, 177815)**	**69.4<br>(67.3, 71.5)**	**965605<br>(955787, 975423)**	**388.1<br>(384.1, 392.1)**	**136401<br>(135677, 137125)**	**55.0<br>(54.7, 55.3)**	**1274648<br>(1263527, 1285769)**	**512.5<br>(508.0, 517.0)**
Heart failure	172642<br>(167469, 177815)	69.4<br>(67.3, 71.5)	831645<br>(822075, 841215)	333.7<br>(329.8, 337.6)	77177<br>(76632, 77722)	31.1<br>(30.9, 31.3)	1081464<br>(1070572, 1092356)	434.2<br>(429.8, 438.6)
Abdominal aortic aneurysms	—	—	3665<br>(3391, 3939)	1.5<br>(1.4, 1.6)	—	—	3665<br>(3391, 3939)	1.5<br>(1.4, 1.6)
Atheroembolism	—	—	740<br>(620, 860)	0.3<br>(0.3, 0.3)	—	—	740<br>(620, 860)	0.3<br>(0.3, 0.3)
Atherosclerosis and peripheral artery disease	—	—	—	—	13631<br>(13402, 13860)	5.5<br>(5.4, 5.6)	13631<br>(13402, 13860)	5.5
Hypertension without heart failure	—	—	128775<br>(126609, 130941)	52.3<br>(51.4, 53.2)	45542<br>(45124, 45960)	18.4<br>(18.2, 18.6)	174317<br>(172111, 176523)	70.7<br>(69.8, 71.6)
Cardiac arrest	—	—	780<br>(658, 902)	0.3<br>(0.3, 0.4)	51<br>(37, 65)	<0.1<br>(<0.1, <0.1)	831<br>(708, 954)	0.3<br>(0.3, 0.4)

Abbreviations: ED, emergency department; CI, confidence interval

* Counts and rates represent mutually exclusive terminus events as outlined in Figure S1. Rates are standardized by age to the 2010 U.S. Census Population distribution among adults aged ≥18 years.

† Mortality data represent complete counts of all deaths, therefore, they are not subject to sampling error. However, these data are subject to non-sampling error resulting from potential errors in the death reporting process, therefore, we calculated and report the estimates and standard errors for the mortality counts (See Data S1 for additional information).

Table S4. Post ICD-10-CM transition Million Hearts case definition codes by event category and type

Event Category or Type	Emergency Department Primary ICD-10-CM Codes*	Acute Care Hospitalization Primary ICD-10-CM Codes*	Underlying Cause of Death ICD-10 Codes*
**Acute myocardial infarction**	—	I21	I21
**Stroke**			
Acute ischemic cerebral infarction	—	I63	I63
Acute hemorrhagic stroke	—	I60, I61	I60, I61
Acute, but ill-defined, cerebrovascular disease	—	I67.89[†]	—[‡]
Other cerebrovascular disease deaths	—	—	I62, I64, I67, I69
**Symptomatic precursor conditions**			
Other acute and subacute ischemic heart disease	I20.0[§], I24	I20.0[§], I24	I20.0[§], I24
Stable angina pectoris	I20.1, I20.8, I20.9	I20.1, I20.8, I20.9	I20.1, I20.8, I20.9
Transient ischemic attack	G45.0, G45.1, G45.2, G45.8, G45.9, G46.0, G46.1, G46.2, I67.841, I67.848	G45.0, G45.1, G45.2, G45.8, G45.9, G46.0, G46.1, G46.2, I67.841, I67.848	G45.0, G45.1, G45.2, G45.8, G45.9
**Other cardiovascular disease conditions**			
Heart failure	[I11.0, I13.0][‖], I50	[I11.0, I13.0][‖], I50	[I11.0, I13.0][#], I50
Abdominal aortic aneurysms	—	I71.02, I71.3[**]	I71.3, I71.4[††]
Atheroembolism	—	I75	—[‡‡]

Atherosclerosis and peripheral artery disease	—	—	[I70, I73.9[§§]][||||]
Hypertension without heart failure	—	[I10, I11.9, I12.9, I13.10][||]	[I10, I11.9, I12.9, I13.9][#]
Cardiac arrest	—	I46[##]	I46[##]

Abbreviations: ICD-10-CM, International Classification of Diseases, 9th Revision, Clinical Modification codes; ICD-10, International Classification of Diseases, 10th Revision

* Excludes all cases involving women with any pregnancy-related ICD-10-CM/PCS codes (O00–O9A , Z34, or Z36) or contributing cause of death codes (O00–O99).

† ICD-9-CM code 436 only partially maps to ICD-10-CM/PCS code I67.89. However, the Centers for Medicare and Medicaid Services plans on using I67.89 for tracking purposes relating to acute stroke patient management, so the code was included. ICD-9-CM code 436 is currently rarely used, as coders now use more appropriate specific cerebrovascular disease codes.

‡ ICD-9-CM code 436 does not accurately map to an ICD-10 mortality code (closest code: I64, which is included as "Other cerebrovascular disease deaths").

§ "Unstable angina" (i.e. intermediate coronary syndrome, ICD-9-CM 411.1) does not map to an I24 ICD-10-CM/PCS code, but to I20.0.

|| Excludes people with any chronic kidney disease, stage 5, or end-stage renal disease ICD-10-CM/PCS code of N18.5, N18.6, Z49, Z94.0, Z99.2, or Z91.15.

Excludes people with any chronic kidney disease, stage 5, ICD-10 contributing cause of death code of N18.5.

** Includes only acute abdominal aortic dissection and rupture (not asymptomatic or elective admissions for surgery).

†† "Abdominal aortic aneurysm without mention of rupture" is included because—although not explicitly mentioned—deaths from abdominal aortic aneurysm almost always occurs as the result of rupture.

‡‡ ICD-10 mortality codes for embolism/thrombosis (I74) do not specify the cause of the embolism/thrombosis like ICD-10-CM/PCS codes do, therefore they were excluded.

§§ “Peripheral vascular disease, unspecified” is included, because it best represents deaths attributable to “peripheral artery disease.”

|||| Only deaths attributable to atherosclerosis or peripheral artery disease were included because Million Hearts® activities will directly impact mortality related to these types of cases, but not necessarily ED visits or hospitalizations.

Cardiac events were only included if they had a secondary diagnosis/contributing cause of death described using a Million Hearts® ICD-10-CM/PCS or ICD-10 mortality code.

Figure S1. Detailed Million Hearts Mutually Exclusive Event Calculation Methodology Applied to the 2013 NEDS, NIS and NVSS Data

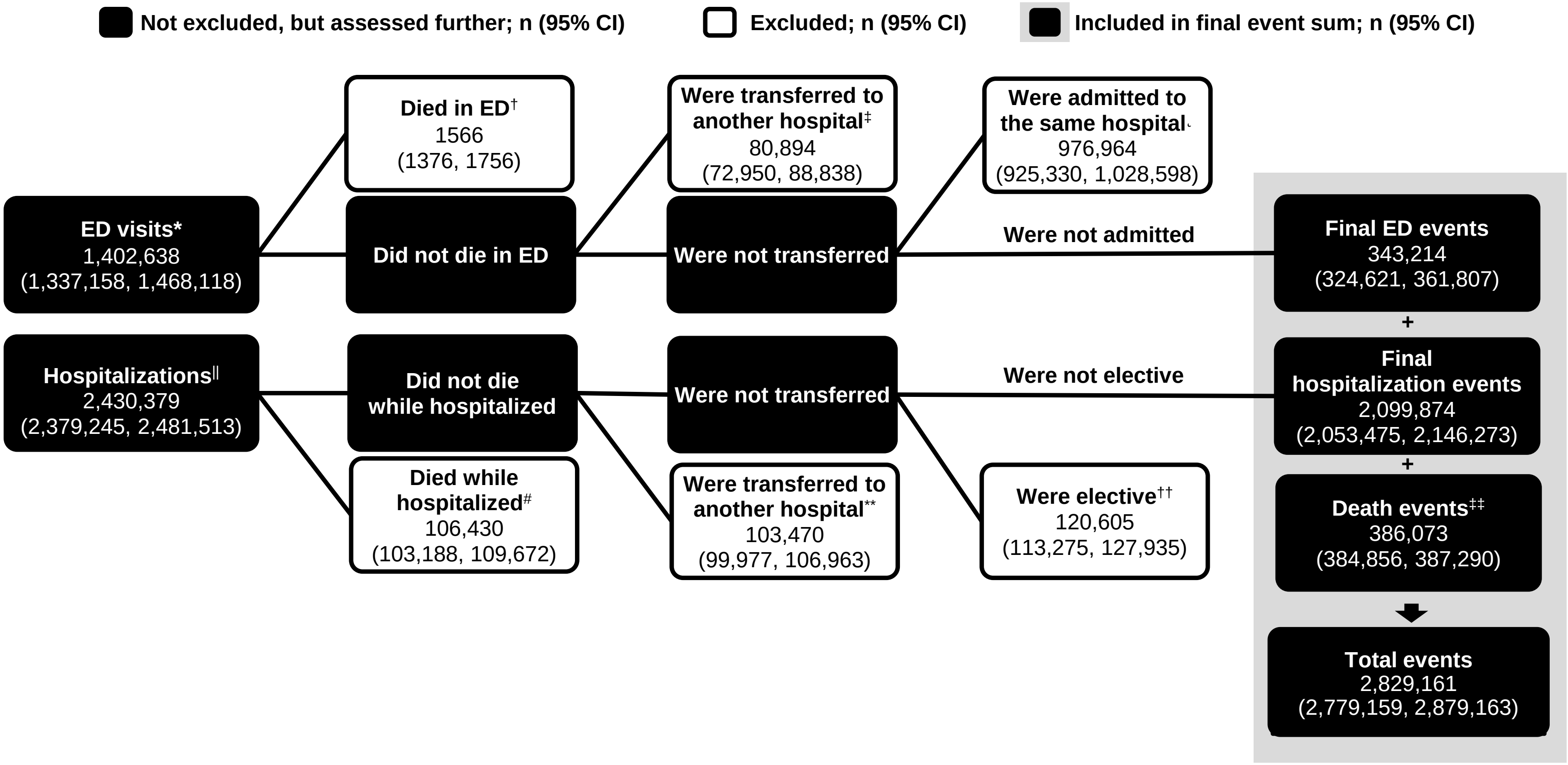

Abbreviations: NEDS, Nationwide Emergency Department Sample; NIS, National Inpatient Sample; NVSS, National Vital Statistics System; CI, confidence interval; ED, emergency department; ICD-9-CM, International Classification of Diseases, 9th Revision, Clinical Modification codes; ICD-10, International Classification of Diseases, 10th Revision

* Measured using the NEDS. Primary ICD-9-CM diagnosis code of: 411, 413, 428, 435, or (402.X1 or 404.X1, excluding 585.5, 585.6, V42.0, V45.1 , or V56). Excludes all cases involving women with any pregnancy-related ICD-9-CM codes (630–677, V22, V23, or V28).

† NEDS variable “died_visit” = 1 (Died in ED).

‡ NEDS variable “disp_ed” = 2 (Transfer to short-term hospital).

§ NEDS variable “disp_ed” = 9 (Admitted as an inpatient to this hospital).

|| Measured using the NIS. Primary ICD-9-CM diagnosis code of: 410.X0, 410.X1, 411, 413, 428, 433.X1, 434.X1, 435, 436, 441.02, 441.3, 445, (401, 402, 403.X0, 404.X0, or 404.X1, excluding 585.5, 585.6, V42.0, V45.1 , or V56), or (427.5 with another previously mentioned code listed as a secondary diagnosis). Excludes all cases involving women with any pregnancy-related ICD-9-CM codes (630–677, V22, V23, or V28).

NIS variable “died” = 1 (Died)

** NIS variable “dispuniform” = 2, except when “dispub04” = 43 (Discharged/transferred to a Federal Health Care Facility); other “dispub04” categories included when “dispuniform” = 2 are: 2 (Discharged/transferred to a Short-Term Hospital for Inpatient Care), 5 (Discharged/transferred to a Designated Cancer Center or Children's Hospital), or 66 (Discharged/transferred to a Critical Access Hospital); Note: Effective prior to 10/1/07, “dispub04” 5 = Discharged/transferred to another type of institution not defined elsewhere

†† NIS variable “Elective” = 1 (Elective admission)

‡‡ Measured using National Vital Statistics death certificate data. Underlying cause of death ICD-10 code of: I20, I21, I24, I50, I60–I69, I71, I71.3, I73.9, I74, (G45, excluding G45.3 and G45.4), (I10, I11, I12.9, I13.0, and I13.9, excluding a contributing cause code of N18.5) or (I46 with another previously mentioned code listed as an contributing cause. Excludes all cases involving women with any contributing cause of death ICD-10 codes (O00–O99).

Figure S2. Example of how the stable and previous trend baselines were established for each sex and age group, men aged 65–74 years

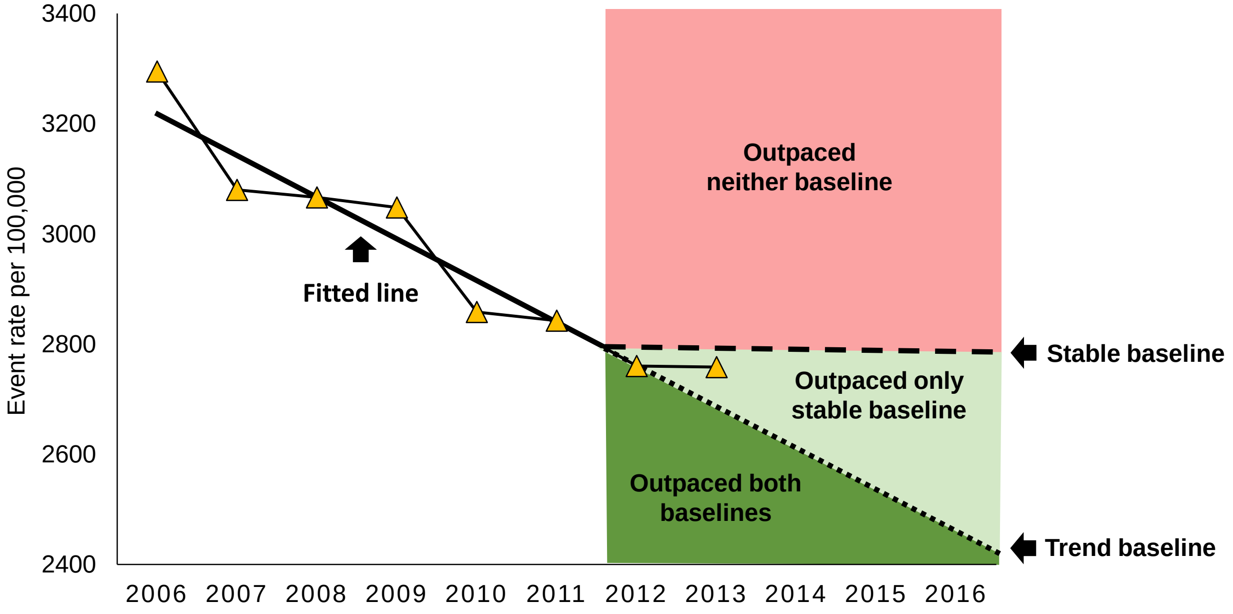

SUPPLEMENTAL REFERENCES:

1. Guide to prevention quality indicators: hospital admission for ambulatory care sensitive conditions. AHRQ Publication No. 02-R0203. Rockville (MD): Agency for Health Care Research and Quality; 2007. Available at: http://www.qualityindicators.ahrq.gov/Downloads/Modules/PQI/V31/pqi_guide_v31.pdf. Accessed September 12, 2014.
2. Outpatient center evaluates stable patients experiencing recent ministroke, leading to timely, comprehensive care and lower costs. Innovations and tools to improve quality and reduce disparities. Agency for Healthcare Research and Quality. US Health and Human Services. Available at https://innovations.ahrq.gov/profiles/outpatient-center-evaluates-stable-patients-experiencing-recent-ministroke-leading-timely. Accessed on July 12, 2015.
3. Yoon PW, Gillespie CD, George MG, Wall HK. Control of hypertension among adults--National Health and Nutrition Examination Survey, United States, 2005-2008. *MMWR. Morbidity and mortality weekly report*. 2012;61 Suppl:19-25.
4. Dalton AR, Vamos EP, Harris MJ, Netuveli G, Wachter RM, Majeed A, Millett C. Impact of universal health insurance coverage on hypertension management: a cross-national study in the United States and England. *PloS One.* 2014;9:e83705.
5. Patient Protection and Affordable Care Act, 42 U.S.C. § 18001 (2010).
6. Mozaffarian D, Benjamin EJ, Go AS, Arnett DK, Blaha MJ, Cushman M, de Ferranti S, Després JP, Fullerton HJ, Howard VJ, Huffman MD, Judd SE, Kissela BM, Lackland DT, Lichtman JH, Lisabeth LD, Liu S, Mackey RH, Matchar DB, McGuire DK, Mohler ER 3rd, Moy CS, Muntner P, Mussolino ME, Nasir K, Neumar RW, Nichol G, Palaniappan L, Pandey DK, Reeves MJ, Rodriguez CJ, Sorlie PD, Stein J, Towfighi A, Turan TN, Virani SS, Willey JZ, Woo D, Yeh RW, Turner MB; American Heart Association Statistics Committee and Stroke Statistics Subcommittee. Heart disease and stroke statistics--2015 update: a report from the American Heart Association. *Circulation.* 2015;131:e29-322.

7. Esposito D, Bagchi AD, Verdier JM, Bencio DS, Kim MS. Medicaid beneficiaries with congestive heart failure: association of medication adherence with healthcare use and costs. *Am J Manag Care.* 2009;15:437-445.
8. Fitzgerald AA, Powers JD, Ho PM, Maddox TM, Peterson PN, Allen LA, Masoudi FA, Magid DJ, Havranek EP. Impact of medication nonadherence on hospitalizations and mortality in heart failure. *J Card Fail.* 2011;17:664-669.
9. Wu JR, Moser DK, Chung ML, Lennie TA. Objectively measured, but not self-reported, medication adherence independently predicts event-free survival in patients with heart failure. *J Card Fail.* 2008;14:203-210.
10. He J, Ogden LG, Bazzano LA, Vupputuri S, Loria C, Whelton PK. Risk factors for congestive heart failure in US men and women: NHANES I epidemiologic follow-up study. *Arch Intern Med.* 2001;161:996-1002.
11. Teunissen LL, Rinkel GJ, Algra A, van Gijn J. Risk factors for subarachnoid hemorrhage: a systematic review. *Stroke.* 1996;27:544-549.
12. National Center for Health Statistics. Vital statistics of the United States: Mortality, 1999 Technical Appendix. 1999. Hyattsville, MD. Available at http://wonder.cdc.gov/wonder/sci_data/mort/mcmort/type_txt/mcmort05/techap99.pdf. Accessed on September 26, 2016.
13. Joinpoint Regression Program, Version 4.2.0.0. April 2015; Statistical Research and Applications Branch, National Cancer Institute.
14. Kim HJ, Fay MP, Feuer EJ, Midthune DN. Permutation tests for joinpoint regression with applications to cancer rates. *Stat Med.* 2000;19:335-51 (correction: 2001;20:655).
